# Parascapular Free Flap for Burnt Hand

**Published:** 2019-02-01

**Authors:** Joseph S. Weisberger, Ramazi O. Datiashvili

**Affiliations:** Division of Plastic & Reconstructive Surgery, Department of Surgery, Rutgers-New Jersey Medical School, Newark

**Keywords:** hand, burn care, fascial flaps, free flaps, parascapular flap

## DESCRIPTION

**Figure 1:**
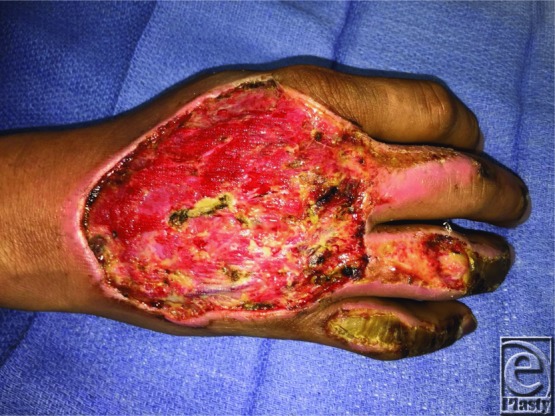
Right hand wound after debridement of necrotic tissue.

**Figure 2:**
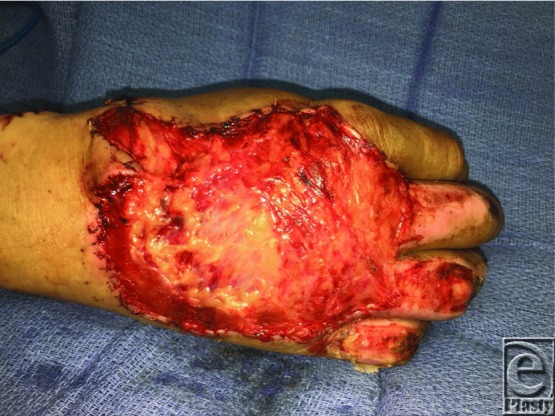
Free parascapular facial flap inset into the wound before skin grafting.

A 40-year-old right-hand-dominant man presented with a third-degree burn to the dorsum of right hand, with exposed tendons, and second-degree burns to his right middle, ring, and small fingers (Fig 1). The patient underwent tangential excision and reconstruction with a parascapular fascial free flap (Fig 2) and split-thickness skin graft.

## QUESTIONS

1. What are the major complications of thermal injury of the hand?
2. What are the reconstructive goals of thermal wound reconstruction of the hand?
3. What are potential options in reconstructing thermal wounds of the hand?
4. What is the anatomy of the parascapular fascial free flap, and why was it a good choice for reconstruction in this case?

## DISCUSSION

Thermal wounds are associated with a myriad of complications including hypertrophic scarring, contracture, pain, and hypersensitivity to contact and temperature.^1^^,^^2^ However, hypertrophic scarring and contracture are of the most concern when dealing with thermal injuries to the hand. Contractures lead to a disruption of function and mobility of the hand and in severe enough cases may warrant amputation of digits. Scarring is the most common cause of morbidity in patients with burn injury and has been reported in more than 50% of burn cases. Studies have shown that the most important factor in determining the development of hypertrophic scarring was time required for wound closure, highlighting the importance of quick and effective reconstructive management of thermal wounds.^3^^,^^4^

The goals of reconstruction following thermal injury to the hand should be to preserve function and mobility, limit long-term sequelae associated with thermal injuries, and restoration of the aesthetic aspect.

While the goals of reconstruction following thermal injury are similar, the approach to management varies on the basis of the extent of injury. In most cases of superficial and partial-thickness burns, it is recommended that wounds be given time to properly heal and the focus of reconstruction should be on the release of tension from contractures and the removal of damaged tissue and hypertrophic scars.^5^ However, surgical intervention may be indicated in full-thickness thermal wounds or deep dermal wounds that have not begun healing in 2 weeks. Studies have shown that early removal of nonviable tissue, followed by subsequent split-thickness skin grafting (STSG), is a viable method for management of full-thickness thermal wound defects of the hand.^6^ Other options in the armamentarium of wound closure following thermal injury to the hand include skin substitutes and various soft tissue transfer. Dermal skin substitutes such as Integra can be used as an adjuvant to STSG in a 2-step process to help reduce scarring in the closure of thermal wounds. Soft tissue free flaps such as fascia, skin, or muscle each have their own distinct role in the management of thermal wound closure. The use of parascapular fascial free flap in combination with a STSG for reconstruction of the complicated soft tissue defect of the hand was reported in the literature.^7^ This technique limits the bulkiness of the flap, provides excellent contouring of the reconstructed area, and allows primary closure of the donor site with its minimal morbidity.

The parascapular free flap is a technique that has been used in the reconstruction of various defects ranging from head and neck to lower extremity defects.^8^ This flap can be harvested on the basis of the vascular supply of the descending branch of the circumflex scapular artery. In this case, the flap was harvested from the left side of the trunk with an 18-cm zigzag incision starting from the posterior aspect of the left axilla, with skin flaps raised on both sides of the incision. The parascapular fascia was identified and mobilized along with the vascular pedicle consisting of the transverse branch of the circumflex scapular artery and vein. The artery of the flap was anastomosed in an end-to-end fashion with the radial artery at the anatomic snuff box, and the vein was anastomosed with the cephalic vein.

**Figure 3:**
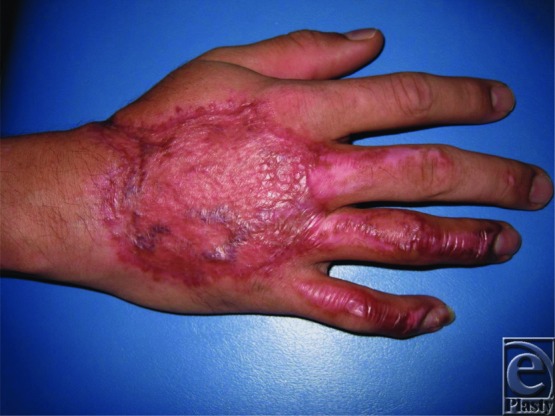
Right hand 2 months post–skin graft.

**Figure 4:**
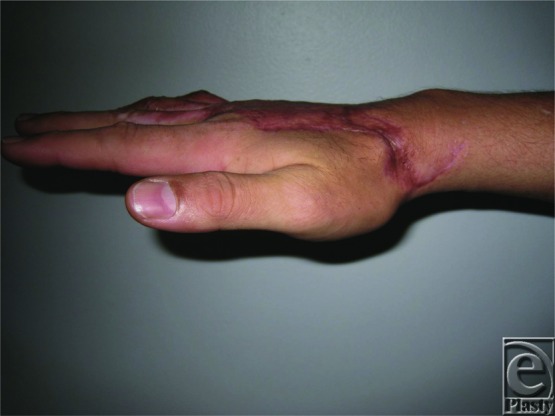
Patient exhibiting full range of extension in all fingers.

**Figure 5:**
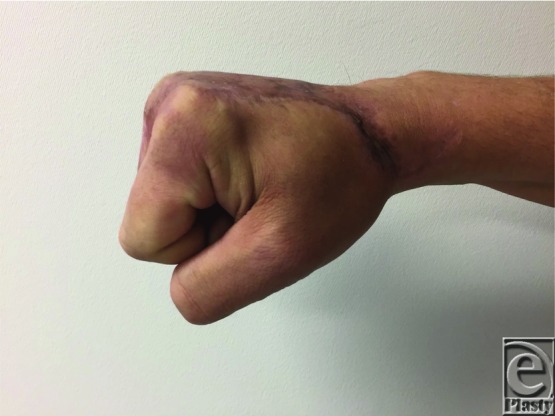
Patient exhibiting full range of flexion of all fingers.

**Figure 6:**
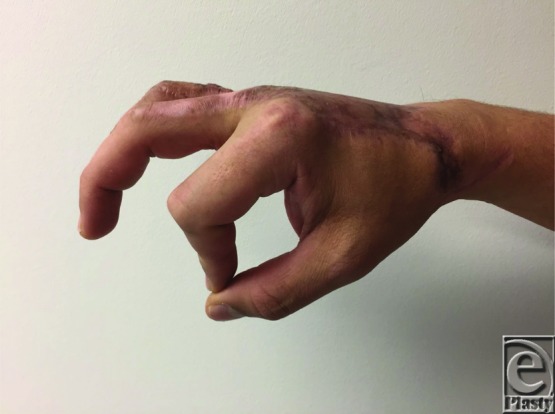
Patient exhibiting pincer grasp.

The hands are involved in close to 80% of all thermal injuries and can result in potentially devastating complications to hand function and mobility. Understanding the potential complications, reconstructive goals, management options, and their complications is essential for optimizing patient outcomes. In this case, the successful full-thickness thermal injury wound closure was accomplished with the early excision of the necrotic tissues and use of parascapular fascial free flap in addition to STSG. Closure of the partial-thickness dermal wounds of the digits was accomplished with the aid of dermal substitutes. Satisfactory aesthetic and functional restoration was achieved (Figs 3–6).
